# Deep learning model can improve the diagnosis rate of endoscopic chronic atrophic gastritis: a prospective cohort study

**DOI:** 10.1186/s12876-022-02212-1

**Published:** 2022-03-23

**Authors:** Quchuan Zhao, Tianyu Chi

**Affiliations:** Department of Gastroenterology, Xuanwu Hospital of Capital Medical University, 45 Chang-chun Street, Beijing, 100053 China

**Keywords:** Artificial intelligence, Deep learning, Gastroscopy, Chronic atrophic gastritis

## Abstract

**Background and aims:**

Chronic atrophic gastritis (CAG) is a precancerous form of gastric cancer. However, with pathological diagnosis as the gold standard, the sensitivity of endoscopic diagnosis of atrophy is only 42%. We developed a deep learning (DL)-based real-time video monitoring diagnostic model for endoscopic CAG and conducted a prospective cohort study to verify whether this diagnostic model could improve the diagnosis rate of endoscopic CAG compared with that of endoscopists.

**Methods:**

A U-NET network was used to build a real-time video monitoring diagnostic model for endoscopic CAG based on DL. We enrolled 431 patients who underwent gastroscopy from October 1, 2020, to December 1, 2020. To keep the baseline data of enrolled patient uniform and control for confounding factors, we applied a paired design and included the same patients in both the DL and the endoscopist group.

**Results:**

The DL model improved the diagnosis rate of endoscopic CAG compared with that of endoscopists. Compared with diagnoses by endoscopists, the proportions of moderate and severe CAG in the atrophy patients diagnosed by the DL model were significantly larger, the proportion of “type O” CAG was significantly larger, the number of atrophy sites found was significantly increased, and the number of biopsies was significantly decreased. Compared with diagnoses by endoscopists, in the atrophic lesions diagnosed by the DL model, the proportions of severe atrophy and severe intestinal metaplasia were significantly increased.

**Conclusions:**

Our study suggested the DL model could improve the diagnosis rate of endoscopic CAG compared with that of endoscopists.

*Trial registration*: ChiCTR2100044458, 18/03/2020.

## Introduction

Gastric cancer is a malignant tumor originating from the gastric mucosal epithelium. It is one of the major diseases that endangers the health of Chinese people. China is a country with high morbidity from gastric cancer, ranking first in the morbidity of malignant tumors of the digestive system. Every year, there are approximately 400,000 new cases of gastric cancer in China and approximately 350,000 deaths. Both new cases and deaths account for 40% of all gastric cancer cases worldwide. Reducing the morbidity and mortality of gastric cancer in China is a major public health problem that must urgently be solved [1].

Chronic atrophic gastritis (CAG) is a precancerous form of gastric cancer. The prevalence of CAG is high in China. The prevalence of CAG in areas with high morbidity of gastric cancer is higher than in areas with low morbidity of gastric cancer, while the diagnosis rate under endoscopy is low, ranging from only 17.7% to 39.8%. Early detection, early diagnosis and early treatment of CAG are effective means to prevent gastric cancer. However, with pathological diagnosis as the “gold standard”, the sensitivity of endoscopic diagnosis of atrophy is only 42%, while the specificity is 91% [2]. Therefore, how to improve the diagnostic rate of endoscopic CAG and the coincidence rate of pathological diagnosis has always been a popular issue receiving clinical attention.

In recent years, artificial intelligence (AI) has been making breakthroughs in the field of image recognition, especially the emergence of deep learning (DL), which causes the extraction of data features to eliminate the limitations of artificial extraction, which is inefficient and incomplete, bringing a revolutionary impetus to the research and development of artificial intelligence [3]. Although the application of DL technology combined with digestive endoscopy has become a research hotspot in the field of gastrointestinal medicine, the current application of DL is mostly limited to the retrospective recognition of static endoscopic images, and there remains a lack of research on real-time video monitoring diagnosis during endoscopy [4, 5].

Our team developed a DL-based real-time video monitoring diagnostic model for endoscopic CAG and conducted a prospective cohort study to verify whether this diagnostic model could improve the diagnosis rate of endoscopic CAG compared with that of endoscopists.

## Methods

### Sample size calculation

PASS 15 (NCSS, LCC., Kaysville, Utah) was used to calculate the sample size for the cohort study, using the operation procedure: Proportion → Two Independent Proportions → Test (Inequality) → Tests for Two Proportions (Ratios). Previous studies have shown that the diagnosis rate of endoscopic CAG ranges from 17.7% to 39.8% [2, 6]. In our review, we found that the average diagnosis rate of endoscopic CAG for endoscopists who have managed more than 10,000 cases in the endoscopy center of our hospital over the last ten years is approximately 26%. Setting α = 0.05 and β = 0.1, it is assumed that the DL model can improve the diagnosis rate by 50%, that is, the DL model/endoscopist RR = 1.5. According to the estimation of the minimum sample size required, the sample sizes of the DL diagnostic model group and the endoscopist diagnostic group were equal. The DL diagnostic model group and the endoscopist diagnostic group required 263 samples.

We planned to use patients in the cohort to conduct a nested case–control study to verify the sensitivity, specificity and other diagnostic evaluation indices of the DL diagnostic model for CAG. The operational process was as follows. Proportions → One Proportion → Confidence Interval → Confidence Interval for One Proportion. According to the guidelines, with pathological diagnosis as the "gold standard", the sensitivity and specificity of endoscopic diagnosis of atrophy are only 42% and 91%, respectively. We assumed that the DL model could improve the sensitivity by 50%, and we set α = 0.05 and the confidence interval = 10%. According to the estimation of the minimum sample size required, the sample sizes of the chronic atrophic gastritis group and chronic nonatrophic gastritis (CNAG) group were equal. The CAG group and the CNAG group required 93 samples.

### Study design and participants

We performed a prospective cohort study. To keep the baseline data of enrolled patients uniform and to control for confounding factors, we applied a paired design and included the same patients in both the DL group and the endoscopist group. The operational process was as follows. According to the guidelines, 3 biopsies were routinely obtained from the gastric antrum, gastric angle and gastric body in each patient during endoscopic operation, and if necessary, more biopsies were obtained from the suspected atrophy site (endoscopist group). Olympus GIF-HQ290 was used to perform gastroscopy for patients, and narrow band imaging (NBI) technique was used to improve the accuracy of endoscopic diagnosis. Synchronous with the endoscopist's observation, the DL model also marked suspicious atrophy sites in the real-time video monitoring, and finally, the assistant told the endoscopist to biopsy the suspicious atrophy sites labeled by the DL model (DL group). If the suspected atrophy site labeled by the DL model overlapped with the suspected atrophy site observed by the endoscopist, there was no need for another biopsy.

We enrolled 431 patients aged 18 years old or older who underwent gastroscopy at the center for digestive endoscopy in our hospital and who volunteered to participate in this study from October 1, 2020, to December 1, 2020. This study protocol was approved by the ethics committee of Xuanwu Hospital of Capital Medical University. Informed consent was obtained from each patient included in the study.

The exclusion criteria were as follows: (1) patients who could not tolerate gastroscopy and did not complete the procedure; (2) patients found with lesions other than chronic gastritis, such as peptic ulcers or malignancies of the digestive tract during gastroscopy; (3) patients with hypertension, diabetes, coronary heart disease, or cerebrovascular disease; (4) patients with contraindications to biopsy, such as the taking of anticoagulant and antiplatelet drugs; and (5) patients who requested withdrawal from the study during gastroscopy.

### Diagnosis of chronic atrophic gastritis

The endoscopic operation in this study was performed by more than 10,000 experienced endoscopists who had performed gastroscopy. According to the guidelines, a pathological biopsy of chronic gastritis showing atrophy of the inherent glands can lead to a diagnosis of CAG, regardless of the number or degree of atrophy of the biopsy specimen. The severity of CAG can be divided into mild, moderate and severe according to the pathological conditions or C type and O type according to the range of lesions [2, 6].

### Deep learning-based real-time video monitoring diagnostic model for endoscopic chronic atrophic gastritis

DL is an improvement of artificial neural networks, which are composed of more layers of neural nodes, with the higher layers obtaining more abstract information for data prediction. Currently, DL has had great success in the field of computer vision. Among these successes, the most representative convolutional neural network (CNN) consists of a series of convolution layers, pooling layers and fully connected layers. Similar to low-level visual processing in the human brain, the low-level convolution layer extracts image features, such as lines or circles that might represent straight edges (such as organ detection) or circles (colon polyp detection), followed by higher-order features, such as local and global shapes and texture feature extraction [3]. CNNs must be trained on a large amount of training data, and it is difficult for medical images to provide such large-scale data [7]. However, there is now a network model particularly suitable for biomedical image processing, namely the U-Net network. The main idea of U-Net is to add a network similar to previous networks after pooling layers, in which the pooling operator is replaced by the upsampling operator. Therefore, these layers increase the resolution of the output. For localization, the high-resolution features from the pooling path are combined with the upsampled output. The continuous convolution layer can then be trained to assemble more accurate output based on this information [8]. Since the U-NET network was proposed, it has been widely used in medical image segmentation. Due to its U-shaped structure that combines context information, fast training speed and small amounts of data, it can meet the demands of medical image segmentation [9]. U-Net was first published in MICCAI in 2015 and then became the baseline model for most of the semantic segmentation tasks on medical images. It also inspired a large number of researchers to consider U-shaped semantic segmentation networks. However, an increasing number of semantic segmentation and target detection SOA (state of art) models have begun to focus on and use U-shaped structures in natural image understanding [10].

### Deep learning model training and testing

In this study, the U-NET network was used to build a real-time video monitoring diagnostic model for endoscopic CAG based on DL. According to the pathological diagnosis, 5290 high-quality endoscopic pictures of 1711 patients who underwent gastroscopy in our hospital from August 1, 2019, to August 1, 2020, were labeled by two gastroenterologists with experience in performing gastroscopy in more than 10,000 cases. A total of 4175 pictures of CAG were labeled, including 2389 pictures of mild atrophic gastritis, 977 pictures of moderate atrophic gastritis, and 809 pictures of severe atrophic gastritis. In addition, 1115 pictures of CNAG were labeled. Then, 70% of the pictures were randomly included in the training set, and 30% of the pictures were included in the test set. The accuracy of the model was adjusted by fivefold cross-validation with 3703 endoscopic pictures. After the model training was completed, we tested the model with 1587 endoscopic pictures, and the sensitivity, specificity and accuracy of the model for the diagnosis of CAG were 92.73%, 92.24% and 92.63%, respectively.

### Outcomes

The primary outcome of this study was to verify whether the DL-based diagnostic model for CAG could improve the diagnostic rate of endoscopic CAG compared to that of endoscopists. The severity, lesion range and number of atrophy sites found in CAG patients and the severity, location, intestinal metaplasia degree, inflammatory activity, and *Helicobacter pylori* infection of atrophic lesions were compared between the DL group and endoscopist group.

The secondary outcome was assessed by using patients in the cohort to conduct a nested case–control study and consider pathological diagnosis as the gold standard to assess the diagnostic evaluation indices of the DL model, such as sensitivity, specificity, and accuracy, and to evaluate its consistency with pathological diagnosis.

### Statistical analysis

Continuous variables are expressed as the mean and standard deviation (SD) or median and interquartile range (IQR) for skewed data, and categorical variables are expressed as frequencies (%). Continuous variables were compared using the paired t-test if normally distributed and Wilcoxon’s signed-rank test if not. Categorical variables were compared using the chi-square test or Fisher’s exact test. Using the data for the patients, logistic regression models were fitted using the CAG as the dependent variable. Receiver operating characteristic (ROC) curves were constructed to assess sensitivity, specificity, and respective areas under the curves (AUCs) with 95% CIs.

A two-tailed P-value < 0.05 was considered statistically significant. All of the analyses were conducted using SPSS software, version 23.0 (IBM Corp., Armonk, NY, USA).

## Results

### Study population

**Fig. 1:**
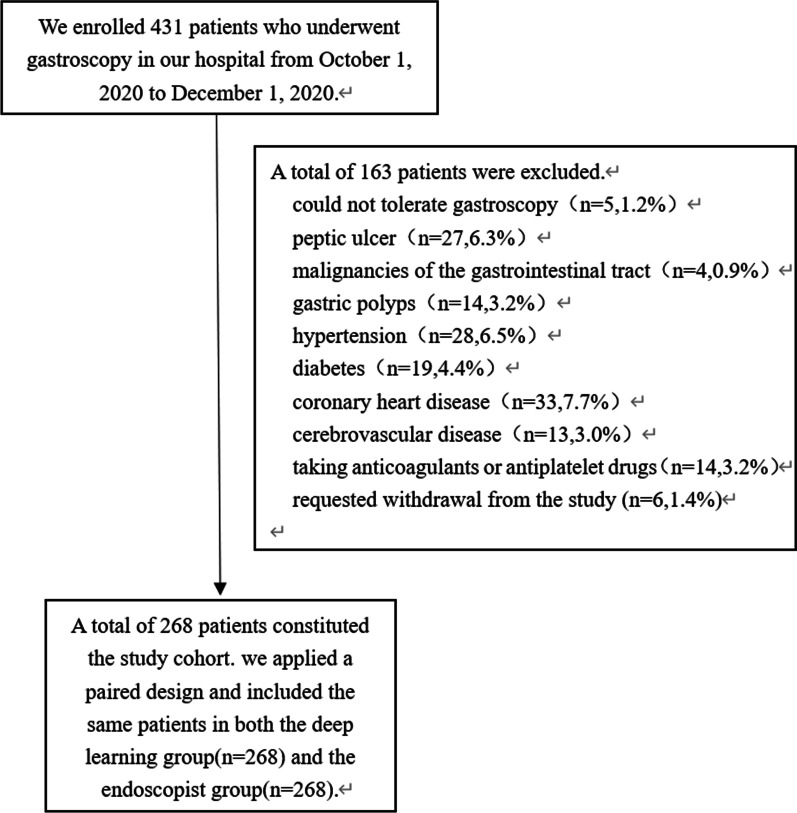
Flow chart of identification of study sample

Figure 1 shows the study flow chart. A total of 431 patients who underwent gastroscopy in the digestive endoscopy center of our hospital were included in the study. A total of 163 patients were excluded. Reasons for exclusion included: 1) patients who could not tolerate gastroscopy and did not complete the procedure (n = 5, 1.2%); 2) patients who were found to have peptic ulcers (n = 27, 6.3%), malignancies of the digestive tract (n = 4, 0.9%), and gastric polyps (n = 14, 3.2%) during gastroscopy; 3) patients with hypertension (n = 28, 6.5%), diabetes (n = 19, 4.4%), coronary heart disease (n = 33, 7.7%), or cerebrovascular disease (n = 13, 3.0%); 4) patients taking anticoagulants or antiplatelet drugs (n = 14, 3.2%); and 5) patients who requested withdrawal from the study during gastroscopy. (n = 6, 1.4%).

A total of 268 patients constituted the study cohort. In a paired design, each patient was in both the DL group (n = 268) and the endoscopist group (n = 268), and the baseline data of the two groups were identical. The baseline characteristics of the sample are presented in Table 1.

**Table 1:** Baseline characteristics of the study cohort

Characteristic	Deep learning group (n = 268) and Endoscopist group(n = 268)
Sex (n, %)	
Male	151 (56.3%)
Female	117 (43.7%)
Age	56.14 ± 14.13
Distribution (n, %)	
< 40 yr	31 (11.6%)
40–59 yr	121 (45.1%)
60–75 yr	94 (35.1%)
> 75 yr	22 (8.2%)
Indication (n, %)	
HP infection	70 (26.1%)
Screening	53 (19.8%)
Diagnostic	145 (54.1%)
Smoking (n, %)	
No	191 (71.3%)
Yes	77 (28.7%)
Drinking (n, %)	
No	187 (69.8%)
Yes	81 (30.2%)
HP infection (n, %)	
No	158 (59.0%)
Yes	110 (41.0%)

*HP Helicobacter pylori*

### Primary outcomes

The DL-based real-time video monitoring diagnostic model for endoscopic CAG improved the diagnosis rate of endoscopic CAG compared with that of endoscopists [35.8% vs. 24.6%, χ^2^ = 7.962, RR = 1.453 (1.117–1.894), *P* = 0.005] (Table 2).

**Table 2:** Risk of primary outcomes in the cohort

Outcome	No. of patients with event	Event rate (%)	Relative risk (95% CI)	χ^2^	*P* Value
CAG				7.962	0.005
DL group	96/268	35.8%	1.453(1.117–1.894)		
Endoscopist group	66/268	24.6%	Reference		

*CAG* Chronic atrophic gastritis, *DL* deep learning

**Fig. 2:**
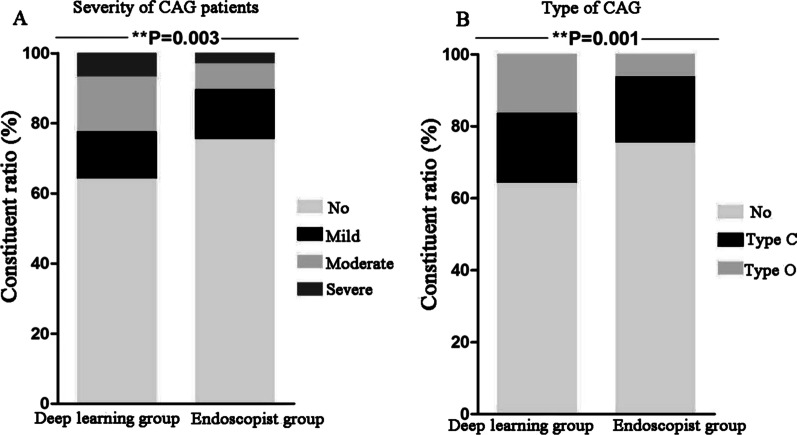
Compared with the endoscopist diagnoses, the proportions of moderate and severe CAG in the atrophy patients diagnosed by the DL model were significantly larger, and the proportion of “type O” CAG was significantly larger

**Fig. 3:**
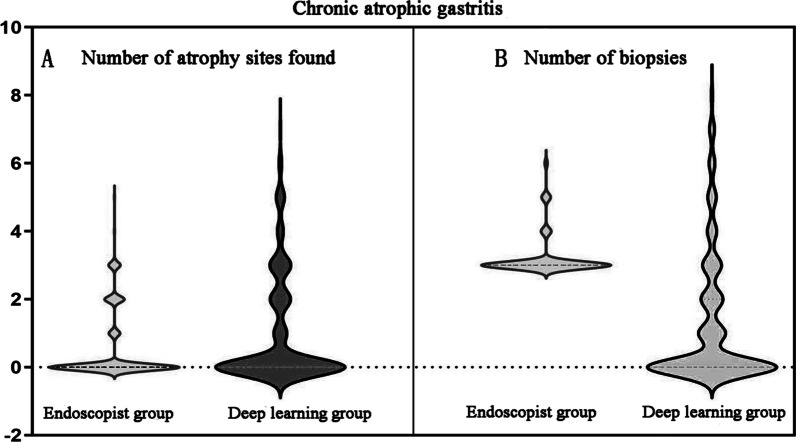
Compared with the endoscopist diagnoses, the number of atrophy sites found was significantly increased and the number of biopsies was significantly decreased

Compared with the endoscopist diagnoses, the proportions of moderate (15.7% vs. 7.5%, χ^2^ = 8.828, *P* = 0.003) and severe (6.7% vs. 3.0%, χ^2^ = 4.042, *P* = 0.044) CAG in the atrophy patients diagnosed by the DL model were significantly larger, and the proportion of “type O” CAG (16.4% vs. 6.3%, χ^2^ = 13.486, *P* < 0.001) was significantly larger. Further, the number of atrophy sites found (1.019 ± 1.613 vs. 0.522 ± 1.000, *t* = −7.026, *P* < 0.001) was significantly increased, the number of biopsies (1.284 ± 1.928 vs. 3.243 ± 0.645, *t* = 18.976, *P* < 0.001) was significantly decreased, and the ratio of the number of atrophy sites found to the number of biopsies [273/344 (79.36%) vs. 140/869 (16.11%), χ^2^ = 439.056, *P* < 0.001] was significantly increased. (Table 3; Figs. 2 and 3).

**Table 3:** The severity, lesion range and number of atrophy sites found in CAG patient

	Deep learning group (n = 268)	Endoscopist group (n = 268)	χ^2^/*t*	*P* Value
The severity of CAG (n, %)			14.113	0.003
No	172 (64.2%)	202 (75.4%)	7.962	0.005
Mild	36 (13.4%)	38 (14.2%)	0.063	0.802
Moderate	42 (15.7%)	20 (7.5%)	8.828	0.003
Severe	18 (6.7%)	8 (3.0%)	4.042	0.044
Type of CAG (n, %)			14.446	0.001
No	172 (64.2%)	202 (75.4%)	7.962	0.005
Type C	52 (19.4%)	49 (18.3%)	0.110	0.740
Type O	44 (16.4%)	17 (6.3%)	13.486	0.000
Number of atrophy sites	1.019 ± 1.613	0.522 ± 1.000	− 7.026	0.000
Number of biopsies	1.284 ± 1.928	3.243 ± 0.645	18.976	0.000
Number of atrophy sites/number of biopsies	273/344 (79.36%)	140/869 (16.11%)	439.056	0.000

*CAG* Chronic atrophic gastritis

**Fig. 4:**
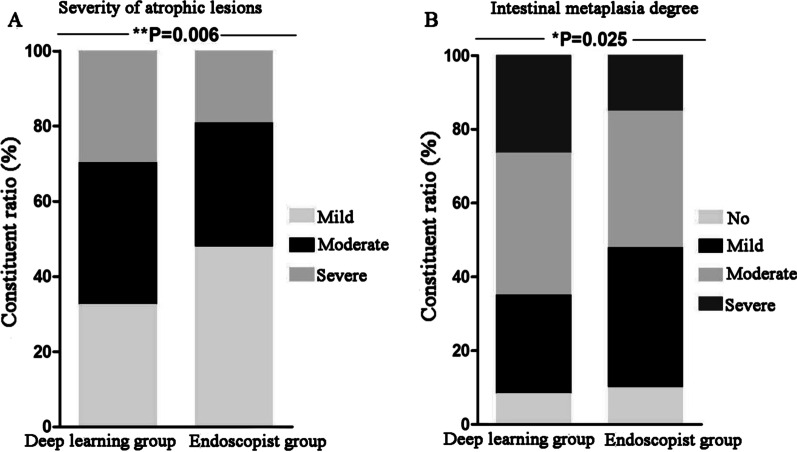
Compared with the endoscopist diagnoses, in the atrophic lesions diagnosed by the DL model, the proportion of severe atrophy and the proportion of severe intestinal metaplasia were significantly increased

Compared with the endoscopist diagnoses, in the atrophic lesions diagnosed by the DL model, the proportion of severe atrophy (29.7% vs.19.3%, χ^2^ = 5.168, *P* = 0.023) and the proportion of severe intestinal metaplasia (26.4% vs. 15.0%, χ^2^ = 6.861, *P* = 0.009) were significantly increased, and the difference was statistically significant (Table 4; Fig. 4).

**Table 4:** The severity, location, intestinal metaplasia degree, inflammatory activity, and *Helicobacter pylori* infection of atrophic lesions

	Deep learning group (n = 273)	Endoscopist group (n = 140)	χ^2^	*P* Value
Severity of atrophic lesions (n, %)			10.128	0.006
Mild	89 (32.6%)	67 (47.9%)	9.164	0.002
Moderate	103 (37.7%)	46 (32.9%)	0.952	0.329
Severe	81 (29.7%)	27 (19.3%)	5.168	0.023
Location of atrophic lesions (n, %)			2.610	0.456
Gastric antrum	137 (50.2%)	61 (43.6%)		
Gastric angle	79 (28.9%)	45 (32.1%)		
Gastric body	36 (13.2%)	18 (12.9%)		
Gastric fundus	21 (7.7%)	16 (11.4%)		
Intestinal metaplasia degree (n, %)			9.364	0.025
No	23 (8.4%)	14 (10.0%)	0.281	0.596
Mild	73 (26.7%)	53 (37.9%)	5.395	0.020
Moderate	105 (38.5%)	52 (37.1%)	0.068	0.794
Severe	72 (26.4%)	21 (15.0%)	6.861	0.009
Inflammatory activity (n, %)			1.493	0.222
No	171 (62.6%)	79 (56.4%)		
Yes	102 (37.4%)	61 (43.6%)		
HP infection (n, %)			0.261	0.609
No	106 (38.8%)	58 (41.4%)		
Yes	167 (61.2%)	82 (58.6%)		

*HP Helicobacter pylori*

Table 5 summarizes the outcomes according to the DL group and endoscopist group. The two logistic regression models showed that DL improved the patients’ diagnostic rate of endoscopic CAG [OR = 1.817 (1.223–2.699), *P* = 0.003].

**Table 5:** Logistic regression analysis (deep learning group vs. endoscopist group) in the cohort

CAG	S.E	Wald	Odds ratio (95% CI)	*P* value
Unadjusted	0.191	7.892	1.708 (1.176–2.482)	0.005
Adjusted for all covariates^a^	0.202	8.747	1.817 (1.223–2.699)	0.003

*CAG* Chronic atrophic gastritis

^a^All variables in Table 1 included as covariates. For model with CAG

### Secondary outcomes

A nested case–control study was conducted with this cohort, and the baseline characteristics of the patients in the CAG group and the CNAG group were basically identical, except for *Helicobacter pylori* infection, as well as sex, age, age distribution, examination indications, smoking and alcohol consumption (Table 6).

**Table 6:** Baseline characteristics of the nested case–control study

Characteristic	CAG group (n = 109)	CNAG group (n = 159)	χ^2^/*t*	*P* value
Sex (n, %)			0.249	0.618
Male	63 (57.8%)	87 (54.7%)		
Female	46 (42.2%)	72 (45.3%)		
Age	56.41 ± 14.52	55.95 ± 13.93	− 0.263	0.793
Distribution (n, %)			1.607	0.658
< 40 yr	15 (13.8%)	16 (10.1%)		
40–59 yr	45 (41.3%)	76 (47.8%)		
60–75 yr	39 (35.8%)	55 (34.6%)		
> 75 yr	10 (9.2%)	12 (7.5%)		
Indication (n, %)			0.190	0.909
HP infection	30 (27.5%)	40 (25.2%)		
Screening	21 (19.3%)	32 (20.1%)		
Diagnostic	58 (53.2%)	87 (54.7%)		
Smoking (n, %)			1.408	0.235
No	82 (75.2%)	109 (68.6%)		
Yes	27 (24.8%)	50 (31.4%)		
Drinking (n, %)			0.310	0.578
No	74 (67.9%)	113 (71.1%)		
Yes	35 (32.1%)	46 (28.9%)		
HP infection (n, %)			11.238	0.001
No	51 (46.8%)	107 (67.3%)		
Yes	58 (53.2%)	52 (32.7%)		

*CAG* Chronic atrophic gastritis, *CNAG* chronic nonatrophic gastritis, *HP Helicobacter pylori*

**Fig. 5:**
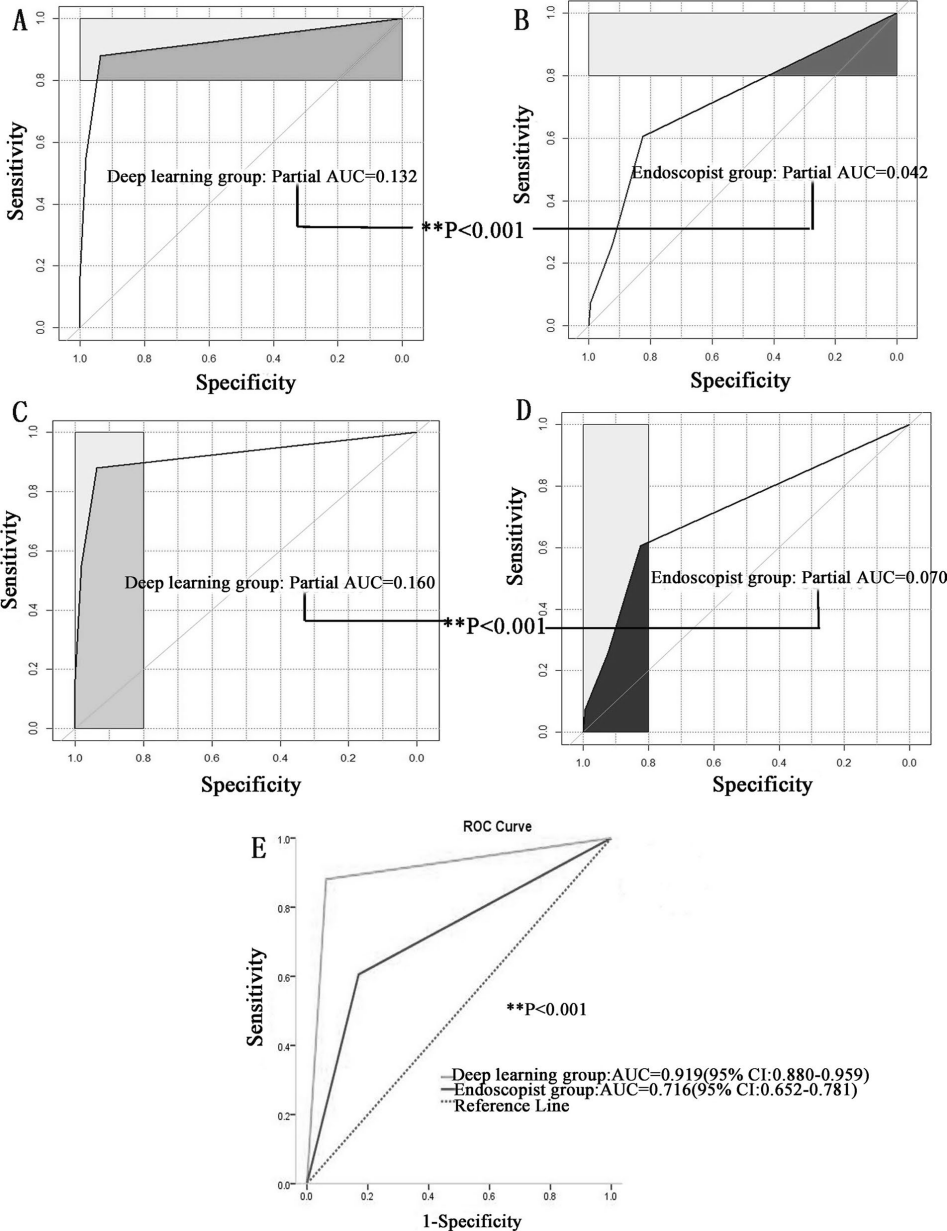
Considering pathological diagnosis as the gold standard, the diagnostic evaluation indices and the evaluation of consistency with pathological diagnosis in the DL group were better than those in the endoscopist group

Considering pathological diagnosis as the gold standard, the diagnostic evaluation indices and the evaluation of consistency with pathological diagnosis in the DL group were better than those in the endoscopist group: sensitivity (88.07% vs. 60.55%) and specificity (93.71% vs. 83.02%), positive predictive value (PV+) (90.57% vs. 70.97%), negative predictive value (PV−) (91.98% vs. 75.43%), accuracy (91.42% vs. 73.88%), Youden index (YI) (81.78% vs. 43.57%), odd product (OP) (110.03 vs. 7.50), positive likelihood ratio (LR+) (14.00 vs. 3.57), negative likelihood ratio (LR−) (0.13 vs. 0.48), AUC (95% CI) [0.919 (0.880–0.959) (*P* < 0.001) vs. 0.716 (0.652–0.781) (*P* < 0.001)] and Kappa [0.821 (*P* < 0.001) vs. 0.446 (*P* < 0.001)] (Table 7; Fig. 5).

**Table 7:** Diagnostic evaluation indices and the evaluation of consistency with pathological diagnosis in the deep learning group and endoscopist group

	Deep learning group	Endoscopist group
Sensitivity	88.07%	60.55%
Specificity	93.71%	83.02%
PV+	90.57%	70.97%
PV−	91.98%	75.43%
Accuracy	91.42%	73.88%
Youden index	81.78%	43.57%
Odd product	110.03	7.50
LR+	14.00	3.57
LR−	0.13	0.48
AUC (95% CI)	0.919 (0.880–0.959) (*P* < 0.001)	0.716 (0.652–0.781) (*P* < 0.001)
Kappa	0.821 (*P* < 0.001)	0.446 (*P* < 0.001)

*PV*+ positive predictive value, *PV− *negative predictive value, *LR*+ positive likelihood ratio, *LR− *negative likelihood ratio

## Discussion

As a precancerous form of gastric cancer, CAG is a disease of focus that presents difficulties to endoscopists. Early detection and diagnosis of CAG can prevent the formation of gastric cancer to a certain extent, but the difficulty of diagnosis and the rate of missed diagnosis have brought great challenges to endoscopists [11]. China is a country with a large population. Endoscopists often need to perform gastroscopy on 10–20 patients within half a working day and deliver the endoscopic diagnosis report to patients immediately. Therefore, improving the accuracy of endoscopic diagnosis is a challenge that every endoscopists must face. Previous studies have shown that the diagnosis rate of endoscopic CAG varies greatly in different hospitals in the same and different regions, fluctuating from 17.7% to 39.8%, and the sensitivity of endoscopic diagnosis of atrophy is only 42% [2]. Therefore, it is particularly important to improve the diagnosis rate of endoscopic CAG. According to the “Consensus of Chronic Gastritis of China”, the endoscopic manifestations of CAG are red and white mucosa, mainly white mucosa; the folds flatten or even disappear, and some mucosal vessels are exposed, which can be accompanied by mucosal granules or nodules [6]. However, in clinical practice, identifying atrophy is mainly based on the subjective understanding of endoscopists, and it depends on their understanding of the guidelines, previous operating experience, the standard training level of the hospital and other factors. Therefore, there are many uncertainties and great differences. How to perform consistent and accurate early detection and diagnosis of CAG by each endoscopist has always been a difficult problem that clinical guidelines have been attempting, but have been unable, to solve.

The emergence of artificial intelligence, especially DL, has provided a better solution to this problem. DL is currently a research hotspot in the field of machine learning. It has shown excellent performance in image recognition and other fields [3, 12]. Its combined application with digestive endoscopy has become a popular research topic, especially for upper gastrointestinal diseases [13, 14]. Currently, the main research directions include DL auxiliary detection of Barrett's esophagus, esophageal cancer, gastric cancer, *Helicobacter pylori* infection and anatomical sites, especially for early cancer. In the upper gastrointestinal endoscopy application field, simple reliance on endoscopists for endoscopic diagnosis still has many limitations and difficulties, such as the diagnosis and differential diagnosis of early malignant tumors (early esophageal cancer and gastric cancer, etc.); approximately 10% of malignant lesions might be missing, but computer-aided diagnosis (CAD) can help endoscopists to accurately detect and screen for early cancer. Some scholars have established a CAD system capable of automatic detection of early gastric cancer using a large number of traditional endoscopic pictures and a convolutional neural network in the DL algorithm. It has high speed of lesion recognition and sensitivity of 92%, indicating that the CAD system with this algorithm as the core has strong clinical diagnostic ability [15]. China's independent research and development of cancer of upper gastrointestinal endoscopy AI auxiliary diagnosis system performance are good. A multicenter, randomized, controlled trial indicated that the system of upper gastrointestinal cancer diagnosis has high accuracy, sensitivity and specificity; the sensitivity can be close to the diagnosis of endoscopic experts and has been established as a preliminary application in clinical endoscopic examination [14]. While many scholars have focused on early cancer of the upper gastrointestinal tract, our study focused on early lesions of “early gastric cancer”—“chronic atrophic gastritis” —to “move forward the threshold” and more effectively reduce the occurrence of gastric cancer and monitor the development of gastric cancer. Some studies have achieved high sensitivity recognition of gastric precancerous lesions, such as polyps, ulcers and erosions, by simplifying many parameters of the neural network under the training of a small data set, with sensitivity of up to 88.9% [16]. Research on AI auxiliary detection for the diagnosis of CAG has also gradually developed [17]. A study suggested that the accuracy, sensitivity and specificity of a convolutional neural network for the diagnosis of CAG were 0.942, 0.945 and 0.940, respectively, which were higher than those of endoscopic experts, and the mild, moderate and severe atrophic gastritis detection rates were 93%, 95% and 99%, respectively [18]. However, the data used for training and validation of the model in most of the studies were retrospective, endoscopic static pictures, and the data were artificially preliminarily screened; thus, they lacked prospective research results [19]. Prospective studies have also focused on the recognition of static pictures [20], while the recognition of real-time video monitoring has been less common [17]. Therefore, we conducted a study to apply the DL-based diagnostic model for CAG to real-time video monitoring in gastroscopy. The study suggested that, compared with endoscopist diagnoses, the DL model could effectively improve the diagnosis rate of endoscopic CAG.

Considering that previous studies of CAD-assisted diagnosis of CAG were mostly limited to retrospective static picture recognition studies [17, 18], our study is a good extension of these previous studies. We developed a DL-based diagnostic model for CAG that can be applied for real-time video monitoring in gastroscopy, and we conducted a prospective cohort study, which suggested that this diagnostic model can improve the diagnostic rate of endoscopic CAG compared with endoscopists [35.8% vs. 24.6%, χ^2^ = 7.962, RR = 1.453(1.117–1.894, *P* = 0.005]. Endoscopic examination is highly dependent on the clinical experience and status of endoscopists, especially for the endoscopic diagnosis of CAG. Inexperience with endoscopists and fatigue during operations often lead to missed diagnoses [21]. Our DL model can overcome the above shortcomings. For endoscopist, it can serve as an experienced instructor to remind them of the location of atrophy and improve the diagnosis rate and as an assistant who does not get fatigued, reminding the endoscopist in a timely manner about lesions that might be neglected and noting omissions. Therefore, our diagnostic model could be a powerful means to assist endoscopists in the early detection and diagnosis of CAG.

The study also found that the proportions of moderate and severe atrophy patients diagnosed by the DL model were significantly larger than that of the endoscopist. Among the atrophic lesions diagnosed by the DL model, the proportion of severe atrophic lesions was significantly increased, reflecting that the DL model can more accurately and objectively evaluate the severity of atrophy and avoid the misjudgment of endoscopists regarding the patient's condition. Previous studies have shown that the pathological diagnosis of patients with CAG at the second visit is often worse than that at the first visit, which is considered to be related to most patients being screened during the first visit for gastroscopy and few endoscopic precision examinations being performed [22–24]. The first visit process of gastroscopy is mainly to find the lesions and perform a preliminary evaluation. The nature and severity of the lesions are mainly determined by pathological diagnosis. Even experienced endoscopists will inevitably misdiagnose lesions. The patients at the second visit were subjected to precision endoscopic examination, which can be evaluated based on the pathological results of the first examination, which is more in-depth and accurate than the first examination. The DL model can effectively avoid the above deficiencies, assisting the endoscopist in labeling all atrophic lesions during the first visit for gastroscopy, accurately assessing the severity of lesions, and performing accurate biopsy.

Among the atrophic lesions diagnosed by the DL model, the proportion of severe intestinal metaplasia was significantly larger than that found by the endoscopist. The range and subtypes of intestinal metaplasia have important value in predicting the risk of gastric cancer, and the severity of intestinal metaplasia determines the assessment of gastric cancer risk staging by Operative Link for Gastric Intestinal Metaplasia Assessment (OLGIM) [11, 25]. The manifestations of intestinal metaplasia under gastroscopy are different, including light yellow nodular type, porcelain white small nodular type, fish-scale type, diffuse type, etc. [2]. These findings are heavily dependent on the operation experience and operation status of the endoscopist, which can lead to missed diagnoses. However, the DL model does not have the above problems, so it can easily and consistently find all types of intestinal metaplastic lesions that the model has learned and evaluate their severity.

In our study, it was found that, compared with endoscopist diagnoses, the proportion of patients diagnosed with “type O” CAG was significantly increased by the DL model, while the proportion of “type C” CAG was not significantly different. Our results were consistent with the conclusions drawn from previous studies. Because the observation of the gastric fundus and gastric body requires turning over the head of the gastroscope, the observation angle and light will be significantly changed compared with frontal observation sites, such as the gastric antrum and gastric angle. Endoscopists must readjust to the angle and light, and any mistake could lead to missed diagnoses [26, 27]. However, the DL model can effectively overcome the above difficulties. The observation angle and light change have almost no influence on its stability, and it can effectively detect atrophic lesions of the gastric fundus and gastric body.

Compared with endoscopist diagnoses, DL model showed that the number of atrophy sites found was significantly increased, the number of biopsies was significantly decreased, and the ratio of the number of atrophy sites found to the number of biopsies was significantly increased. The “Consensus of Chronic Gastritis of China” points out that histopathology is very important for the diagnosis of chronic gastritis, and biopsy should be performed according to the endoscopic conditions and needs. For clinical diagnosis, it is recommended to obtain 2 to 3 pieces of tissue from the gastric antral, gastric angle and gastric body for histopathology, and additional biopsy must be performed of suspicious lesions, the purpose of which is to prevent missed diagnoses through multisite biopsy [2, 28]. However, multisite biopsy is prone to excessive mucosal damage and prolonged operation times, leading to an increased risk of intraoperative and postoperative bleeding and cardiovascular and cerebrovascular complications. The DL model can overcome the above shortcomings, not only improving the number of atrophic lesions detected but also reducing the number of blind-spot biopsies; it not only reduces mucosal injury but also reduces the operation time of gastroscopy. It not only reduced the operation burden and pressure on endoscopists but also reduced the duration of operation-related pain and risks to patients.

We also used the cohort to conduct a nested case–control study. Considering pathological diagnosis as the gold standard, the diagnostic evaluation indices and the evaluation of consistency with pathological diagnosis in the DL group were better than those in the endoscopist group. Therefore, the DL model of CAG has better ability to detect CAG and identify CNAG. Those diagnosed as positive by the DL model have a greater probability of having CAG. Those diagnosed as negative by the DL model have a greater probability of having CNAG. The AUC for the DL model was > 0.9, indicating high diagnostic accuracy. The Kappa value for the DL model was > 0.8, showing good consistency with the pathological diagnosis.

There are some limitations of our study. First, because it was an exploratory study, our exclusion criteria were strict to avoid risks to patients. We excluded patients with endoscopic lesions other than chronic gastritis, such as peptic ulcers and gastrointestinal malignant tumors, as well as patients with chronic medical histories, such as hypertension, diabetes, coronary heart disease and cerebrovascular disease, which resulted in a certain bias in the sample enrolled in the cohort. After obtaining the successful experience of this study, we will include a broader range of patients with chronic gastritis complicated with other endoscopic lesions and with chronic diseases in our future cohorts to further scientifically verify our model. Second, we conducted a nested case–control study with this cohort, considering pathological diagnosis as the gold standard to verify the diagnostic evaluation indices of our model and its consistency with pathological diagnosis. Due to the short study period and insufficiently large cohort, although the sample size met the standards of statistical efficiency, the baseline data of the CAG group and the CNAG group were not completely equal, so the evaluation indices calculated were biased to some extent. In the future, we will continue to expand our cohort, strictly match the baseline data of the CAG group and the CNAG group and recalculate and correct our evaluation indices.. Third, as this is an exploratory study, we conducted the study with a single-center cohort. The enrolled cases were only representative of this region and may have selection bias. In futures stages, we will include additional regions for a multicenter study so as to make our research results more broadly representative.

## Conclusion

Our prospective cohort study suggested that the DL-based real-time video monitoring diagnostic model for endoscopic CAG could improve the diagnosis rate of endoscopic CAG compared with that of endoscopists and could better assist endoscopists in the diagnosis of endoscopic CAG.

## Data Availability

The dataset generated and analyzed during the study is stored in a secure localized database but is available from the corresponding author in an anonymous format on reasonable request.

## Abbreviations

CAG: Chronic atrophic gastritis
DL: Deep learning
AI: Artificial intelligence
CNAG: Chronic nonatrophic gastritis
CNN: Convolutional neural network
ROC: Receiver operating characteristic
CAD: Computer-aided diagnosis
NBI: Narrow band imaging
